# Platycodin D inhibits autophagy and increases glioblastoma cell death via LDLR upregulation

**DOI:** 10.1002/1878-0261.12966

**Published:** 2021-05-02

**Authors:** Sol Ji Lee, Yu‐Jeong Choi, Hyo In Kim, Hyo Eun Moon, Sun Ha Paek, Tai Young Kim, Seong‐Gyu Ko

**Affiliations:** ^1^ Department of Science in Korean Medicine Graduate School Kyung Hee University Seoul Korea; ^2^ Center for Cognition and Sociality Institute for Basic Science Daejeon Korea; ^3^ Department of Neurosurgery Advanced Institute of Convergence Technology (AICT) Cancer Research Institute, and Ischemic/Hypoxic Disease Institute Seoul National University College of Medicine Korea; ^4^ Department of Preventive Medicine College of Korean Medicine Kyung Hee University Seoul Korea

**Keywords:** autophagy, cholesterol, GBM, LDLR, lysosome, platycodin D

## Abstract

Targeting autophagy is a promising therapeutic approach in cancer therapy. Here, we screened 30 traditional herbal medicines to identify novel autophagy regulators and found that *Platycodon grandiflorus* (PG) and platycodin D (PD), a triterpenoid saponin from PG, inhibited autophagy in glioblastoma multiforme (GBM) cells. Mechanistically, PD prevented lysosomal degradation and the fusion between autophagosomes and lysosomes by inducing sequestration of free cholesterol in lysosomes. The autophagy inhibitory effect of PD was mimicked by both genetic and pharmacological inhibition of Niemann‐Pick C1 (NPC1), which exports low‐density lipoprotein (LDL)‐derived cholesterol from lysosomes. Moreover, PD promoted the uptake of exogenous LDL cholesterol via upregulation of LDL receptor (LDLR), leading to further accumulation of cholesterol within lysosomes and GBM cell death. Importantly, these phenomena were more pronounced in LDLR‐overexpressing GBM cells than in normal astrocytes. Finally, blockade of cholesterol uptake by LDLR knockdown reversed the PD‐induced inhibition of autophagy and GBM cell growth. Our study proposes that PD could be a potent anti‐GBM drug by disrupting cholesterol trafficking and autophagy.

## Graphical abstract

Our study demonstrates that platycodin D (PD) increased cholesterol content in lysosomes and prevented lysosomal fusion with autophagosomes in glioblastoma multiforme (GBM) cells. Low cholesterol availability increased LDLR expression and uptake of LDL cholesterol, leading to accumulation of cholesterol in lysosomes and cell death. The anticancer effect of PD correlated with LDLR overexpression in GBM cells, which is in contrast to normal astrocytes expressing low levels of LDLR.

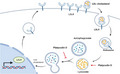

**Abbreviations**
25‐HC: 25‐hydroxycholesterol
ABCA1: ATP‐binding cassette transporter A1
ATG12: autophagy related 12
BafA1: bafilomycin A1
CTSB: cathepsin B
CTSD: cathepsin D
DAPI: 4,6‐diamidino‐2‐phenylindole
DQ Red BSA: Dye‐Quenched BSA
EGFR: epidermal growth factor receptor
ER: endoplasmic reticulum
ERK: extracellular signal‐regulated kinases
GBM: glioblastoma multiforme
IDOL: inducible degrader of LDLR
JNK: c‐JunN‐terminal kinases
LC3: 1A/1B‐light chain 3
LDLR: low‐density lipoprotein receptor
LXR: liver X receptor
NPC: Niemann‐Pick Type C
NS: nonspecific
PD: platycodin D
PFA: paraformaldehyde
PG: *platycodon grandi* *fl* *orus*
RFP: red fluorescent protein
RT: room temperature
SNAREs: soluble *N*‐ethylmaleimide‐sensitive factor attachment protein receptors
SQSTM1/p62: sequestosome 1

## 1 Introduction

Glioblastoma multiforme (GBM) is one of the most common primary brain tumors, which accounts for 17% of all brain tumors. It is characterized by rapid and infiltrative growth associated with high mortality rate [1]. Moreover, despite of advances in therapies including surgery, radiation, and chemotherapy, the prognosis still remains poor. Temozolomide, an alkylating agent, is conventional chemotherapy against GBM. However, this agent has limitation in that it is effective only for a subset of patients and develops resistance [2]. Thus, identification of effective therapeutic approaches to treat GBM patients is urgently demanded. This can be achieved through the understanding of diverse molecular mechanisms that affect GBM growth.

Recent studies have demonstrated that cancer cells have more dependency on autophagy for their survival compared with noncancerous cells [3]. Autophagy is a conserved self‐degradation system that eliminates and recycles cellular components involving unnecessary proteins and damaged organelles [4]. Autophagy flux represents whole dynamic process of autophagy. During autophagy flux, cellular components are engulfed within a double‐membrane vesicle called autophagosome. Then, it fuses with a lysosome to form an autolysosome, which causes the degradation of the sequestered materials using lysosomal proteases [5]. This process is essential for maintaining cellular homeostasis and sustaining cell growth even under extreme conditions [6]. In addition, altered cell metabolism is recognized as a distinct feature of cancer cells and suggested as a potential target for cancer therapy. However, the role of cholesterol metabolism in cancer has not yet been fully explored [7]. Recent studies demonstrated that GBM cells rely more on uptake of exogenous cholesterol than *de novo* biosynthesis for their survival and a drug that deplete the intracellular cholesterol can kill GBM cells [8, 9], and dysregulated cholesterol transport affects autophagy activity, consequently leading to cell death [10, 11].

Medicinal herbs and their natural compounds have been drawn more attentions as alternative therapeutics for the treatment of human diseases owing to their advantages on nontoxicity and fewer side effects. Among them, *Platycodon grandiflorus* (PG) has been widely used in East Asia including Korea, China, and Japan as a functional substance to treat respiratory disease such as asthma, bronchitis, and tonsillitis [12, 13]. In recent studies, PG was further known to have various pharmacological potential in hyperlipidemia, diabetes, obesity, hypertension, and cancer [14]. Platycodin D (PD) is a triterpenoid saponin abundant in PG and a major active compound to exert diverse pharmacological activities. Moreover, it has been increasingly reported that PD exhibits anticancer properties against a wide range of cancer cells through cell proliferation inhibition, cell cycle arrest, and autophagy [15]. However, the effects of PG and PD on GBM have not yet been investigated.

In this study, we observed that PD has an inhibitory effect on the autophagy by blocking autophagosome–lysosome fusion and lysosomal degradative function in GBM cells. We further observed that PD induces the formation of cholesterol deposits in lysosomes and increases uptake of exogenous cholesterol by enhancing cell surface low‐density lipoprotein receptor (LDLR) expression. Moreover, we demonstrated that the PD‐mediated accumulation of cholesterol in lysosomes causes impairment of autophagy flux, leading to GBM cell death. These findings provide new insights into the molecular and functional mechanism by which PD exerts its anticancer activity against GBM.

## 2 Materials and methods

### 2.1 Reagent

The powder of medicinal plants was provided by Hanpoong Pharmaceutical Company (Jeonju, Korea) and dissolved in distilled water. Platycodin D and U18666A were purchased from Cayman Chemical Company [16]. Bafilomycin A1 and rapamycin were purchased from Sigma [16].

### 2.2 Cell culture

Cancer cell lines used in this study including U87MG, U373MG, A549, H358, MCF‐7, HT‐29, and HepG2 were obtained from the Korean Cell Line Bank (KCLB, Seoul, South Korea). Normal human astrocyte was obtained from abm (T0280, Richmond, BC, Canada). Primary GBM cells were obtained at the Seoul National University Hospital under the approval of the Institutional Review Board (IRB, No; H‐0507‐509‐153). U373MG and A549 cells were cultured in RPMI‐1640 and other cells were cultured in DMEM, supplemented with 10% FBS (Corning, Woodland, CA, USA), 100 U·mL^−1^ penicillin–streptomycin (Welgene, Gyeongsan‐si, South Korea) at 37 °C in a humidified incubator containing 5% CO_2_. For cell culture in cholesterol‐free condition, lipoprotein‐depleted FBS (Kalen Biomedical, Germantown, MD, USA) was used.

### 2.3 Cell viability assay

Cells were seeded into 96‐well plate (5 × 10^3^ cells/well) and treated with the serial concentrations of PD for indicated time. After treatments, WST‐1 solution (Dogen, South Korea) was added and incubated for 2 h. Water‐soluble formazan formed in the culture medium was measured by Versa Max microplate reader (Molecular Devices, San Jose, CA, USA) at 450 nm absorbance. The relative cell viability (%) was expressed as a percentage relative to the DMSO‐treated control cells.

### 2.4 Western blot analysis

Cells were washed once with ice‐cold PBS and lysed in ice‐cold lysis buffer (20 mm Tris/HCl, pH 7.5, 150 mm NaCl, 1 mm EDTA, 1 mm Na2EDTA, 1 mm EGTA, 1% NP‐40, 1% sodium deoxycholate, 1 mm Na_3_VO_4_, 1 mm PMSF, and PI cocktails). The extract was cleared by centrifugation at 15 000 *g* for 20 min. The cleared cell lysates were mixed with a proper volume of 5× SDS sample buffer, separated by SDS/PAGE, and transferred to nitrocellulose membranes (Thermo Fisher Scientific, Waltham, MA, USA). After blocking with 5% skim milk in TBST (20 mm Tris/HCl, pH 7.5, 150 mm NaCl, 0.05% Tween 20) for 1 h at room temperature (RT), the membranes were incubated at 4 °C overnight with the following primary antibody: anti‐LC3B, cathepsin B, p‐ULK1 (Ser757), p‐S6K (Thr389), p‐JNK (Thr183/Tyr185), GAPDH (Cell Signaling Technology, Danvers, MA, USA), SQSTM1/p62, cathepsin D, Beclin1, ULK1, ATG7, ATG12, p‐AKT (Ser473), ERK, p‐ERK, JNK1 (Santa Cruz Biotechnology, Dallas, TX, USA), LDLR (Abcam, Cambridge, CB2 0AX, UK), NPC1, and NPC2 (Novus, Centennial, CO, USA). The corresponding horseradish peroxidase‐conjugated secondary antibodies (KPL, Gaithersburg, MD, USA) were incubated 1 h at RT. Antibody–protein complexes were detected using ECL Western Blotting Substrate (Thermo Fisher Scientific, Waltham, MA, USA).

### 2.5 RT‐PCR analysis

Total RNA was isolated using easy‐blue RNA extraction kit (iNtRON Biotech, Seongnam‐si, South Korea) according to the manufacturer's directions. One microgram of total RNA was reverse transcribed using cDNA synthesis kit (TaKaRa, Kusatsu, Japan) according to the manufacturer's instructions. The resulting cDNA was then amplified by PCR in a reaction mixture consisting of 10× buffer, 2.5 mm dNTP mixture, Taq DNA polymerase (TaKaRa, Kusatsu, Japan), and corresponding primer sets. The primer sequences were as follows: p62 (Forward) 5′‐GAACTCCAGTCCCTACAGATGCC‐3′, (Reverse) 5′‐CGGGAGATGTGGGTACAAGG‐3′; LDLR (Forward) 5′‐CAGATATCATCAACGAAGC‐3′, (Reverse) 5′‐CCTCTCACACCAGTTCACTCC‐3′; IDOL (Forward) 5′‐TTGTGGACCTCGTTTCAAGA‐3′, (Reverse) 5′‐GCTGCAGTTCATGCTGCT‐3′; NPC1 (Forward) 5′‐AGCCAGTAATGTCACCGAAAC‐3′, (Reverse) 5′‐CCGAGGTTGAAGATAGTGTCG‐3′; NPC2 (Forward) 5′‐TGGAACTTCGTTATCCGCGA‐3′, (Reverse) 5′‐CACAGAACCGCAGTCCTTGAA‐3′; GAPDH (Forward) 5′‐CGTCTTCACCACCATGGAGA‐3′, (Reverse) 5′‐CGGCCATCACGCCACAGTTT‐3′. The PCR products were loaded onto 1.5% agarose gels and visualized under UV light.

### 2.6 Plasmids, lentivirus production, and infection

Autophagy flux reporter plasmid (pQCXI Puro DsRed‐LC3‐GFP) was a gift from D. Sabatini (Addgene plasmid #31182, Watertown, MA, USA). For gene knockdown, the oligonucleotides that contain shRNA sequence targeting NPC1 (5′‐CCAGGTTCTTGACTTACAA‐3′), LDLR (5′‐CCACTTGTAGGAGATGCAT‐3′), and nonspecific (NS) shRNA plasmid (5′‐CAACAAGATGAAGAGCACCAA‐3′) were cloned into the pLKO.1 puro lentiviral shRNA plasmid. For production of lentiviral particles, the cloned pLKO.1 plasmids were transiently transfected into HEK293T cells with lentiviral packaging plasmid psPAX2 and lentiviral envelope plasmid pMD2.G using Lipofectamine 3000 transfection reagent (Thermo Fisher Scientific, Waltham, MA, USA). The culture supernatants were collected at 24 and 48 h after transfection, combined, and filtered through 0.45‐μm‐pore‐size filters. For lentiviral infection, GBM cells were incubated with the virus‐containing supernatants in the presence of polybrene (Merck, Darmstadt, Germany) at a final concentration of 4 μg·mL^−1^. After incubating for 24 h, the supernatants were replaced with fresh medium and cells were cultured for another 2 days before further experiments.

### 2.7 Cell imaging

For monitoring autophagosome formation or autophagy flux, GBM cells on glass coverslips were transiently transfected with GFP‐LC3 plasmid, followed by treatment with 10 μm PD for 24 h or were infected with lentivirus expressing DsRed‐LC3‐GFP, followed by treatment with 10 μm PD or 1 μm rapamycin for 24 h, respectively. For staining of intracellular p62, GBM cells treated with 10 μm PD or 100 nm BafA1 for 24 h were permeabilized with 0.01% Triton X‐100 in PBS. After blocking in 2% BSA in PBS, the primary antibody against p62 (Santa Cruz, USA) was incubated overnight at 4 °C, followed by further incubation with Alexa594‐conjugated secondary antibody (Invitrogen, Carlsbad, CA, USA) for 1 h at RT. To stain acidic compartments, 50 nm LysoTracker Red DND‐99 (Thermo Fisher Scientific) was added to the medium for 30 min at 37 °C and 5% CO_2_ prior to fixation. The nuclei were labeled with 4, 6‐diamidino‐2‐phenylindole (DAPI; Sigma, St. Louis, MO, USA). The coverslips were finally mounted using faramount mounting medium (Dako, Glostrup, Denmark). The images were examined under a Zeiss laser confocal microscopy (Carl Zeiss, Oberkochen, Germany) and analyzed in zen software (Carl Zeiss).

### 2.8 DQ Red BSA trafficking assay

To examine the changes in lysosomal degradative function in cells, Dye‐Quenched BSA (DQ Red BSA) was used. The red fluorescence of DQ Red BSA is self‐quenched but, upon cleavage in the lysosomes, the fluorescent group is released, producing an increase in fluorescence intensity. Briefly, GBM cells grown on coverslip were treated with DMSO, 10 μm PD, or 100 nm BafA1 for 24 h and subsequently loaded with 10 μg·mL^−1^ DQ Red BSA (Thermo Fisher) in culture medium at 37 °C for 30 min. The cells were then fixed in 4% paraformaldehyde (PFA), and nuclei were stained with 1 μg·mL^−1^ DAPI in 2% BSA solution for 1 min. The stained cells were examined under LSM700 confocal microscope (Carl Zeiss), and the numbers of DQ Red BSA spots were quantified using imagej software (NIH, MD, USA).

### 2.9 Filipin‐III cholesterol staining

The distribution of free cholesterol within cells was visualized by staining cells with filipin‐III, a fluorescent polyene antibiotic that binds specifically to free (unesterified) cholesterol. GBM cells grown on coverslips were treated with 5 μg·mL^−1^ filipin‐III (Cayman, Ann Arbor, MI, USA) in PBS at RT for 30 min after fixation with 4% PFA. The stained cells were then observed under LSM700 confocal microscope (Carl Zeiss), and the images were analyzed with imagej software.

### 2.10 LDL uptake assay

Glioblastoma multiforme cells grown on coverslip were treated with DMSO, 10 μm PD or 1 μm U18666A for 24 h and then incubated with 5 μg·mL^−1^ BODIPY™ FL LDL (Thermo Fisher) for 1 h. Then, the cells were fixed with 4% PFA for 10 min. Nuclei were stained with 1 μg·mL^−1^ DAPI in 2% BSA solution for 1 min. The LDL uptake within cells was observed using LSM700 confocal microscope (Carl Zeiss), and fluorescence intensity was quantified with imagej software.

### 2.11 Measurement of cellular cholesterol contents

Cellular free and cholesteryl ester contents were determined using total cholesterol assay kit (Cell Biolabs, San Diego, CA, USA) according to the instructions. Briefly, 1 × 10^6^ GBM cells treated with 10 μm PD or 1 μm U18666A for 24 h were extracted in 200 µL mixture of chloroform : isopropanol: NP‐40 (7 : 11 : 0.1) in homogenizer. After centrifugation of the extracts for 10 min at 13 000 r.p.m., the organic phase was collected and evaporated at 50 °C, followed by vacuum desiccation for 30 min. The dried pellets were dissolved and incubated with cholesterol reaction reagent solution containing or omitting cholesterol esterase to measure total cholesterol and free cholesterol, respectively. The colorimetric signal was measured using Versa Max microplate reader (Molecular Devices) at 540 nm absorbance. Cholesteryl esters were quantified by subtracting the free cholesterol value from the total cholesterol value, and cholesterol contents were expressed as micrograms of cholesterol per milligram of protein.

### 2.12 Flow cytometry analysis

To analyze the expression of cell surface LDLR, normal human astrocytes and GBM cells were treated with DMSO, 10 μm PD or 1 μm U18666A for 24 h, detached using trypsin/EDTA solution (Gibco, Waltham, MA, USA), and washed twice with 1% BSA in PBS. The cells were then incubated with anti‐LDLR antibodies (Abcam) in PBS containing 5% BSA at 37 °C for 30 min and washed twice with 1% BSA in PBS, followed by incubation with Alexa Fluor 488‐conjugated secondary antibody (Thermo Fisher) at 37 °C for 30 min. The cells were then fixed with 0.5% PFA for 10 min, resuspended in PBS, and analyzed by FACSCalibur flow cytometer (Becton Dickinson, Franklin Lakes, NJ, USA) using cell quest pro software (BD Biosciences, Franklin Lakes, NJ, USA).

### 2.13 Clonogenic assay

Clonogenic assay was performed as described previously [17]. Briefly, 2 × 10^3^ GBM cells were seeded into 6‐well plates. On the next day, the cells were treated with 10 or 20 μm PD. After incubation for additional 7 days, viable colonies were stained with 0.5% crystal violet.

### 2.14 Statistical analysis

Data in this study were presented as the mean ± standard deviation (SD). Statistical analysis was performed using two‐tailed Student's *t*‐test or one‐way analysis of variance (ANOVA) followed by Tukey's *post hoc* test. A *P*‐value less than 0.05 was considered statistically significant (*P < 0.05; **P < 0.01; ***P < 0.001; ****P < 0.0001; NS, not significant).

## 3 Results

### 3.1 Screening herbal medicines for the identification of novel autophagy modulators

To identify novel autophagy modulators from 30 traditional Korean herbal medicines, we examined LC3B‐II levels after treatment with each herbal extracts in two GBM cell lines, U87MG and U373MG cells. Conversion of LC3‐I to LC3B‐II is widely used as a readout for autophagy activity [18]. Western blot analysis showed that LC3B‐II levels were increased when treated with water extracts of *Glycyrrhiza uralensis*, *P. grandiflorus* (PG), and *Rubus coreanus* in U87MG cells and that of PG in U373MG cells (Fig. S1). Next, we evaluated the effect of each extracts on the levels of sequestosome 1 (SQSTM1)/p62, whose increased levels represent autophagy inhibition. The expression level of p62 was increased by the extracts of various herbs including PG in U87MG cells and that of *G. uralensis*, *Scutellaria baicalensis*, and PG in U373MG cells. Collectively, only PG increased the levels of LC3‐II and p62 similarly in both U87MG and U373MG cells. Moreover, none of the herbal extracts did change the expression levels of core autophagy regulators including ULK‐1, Beclin‐1, and ATG12‐ATG5 complex in both GBM cells, though ULK‐1 and ATG12‐ATG5 complex levels were decreased by several herbal extracts in U87MG cells (Fig. S1). Based on these results, we conclude that PG extract may contain bioactive compounds that inhibit autophagy in GBM cells.

### 3.2 Platycodin D is an active compound of PG that inhibits autophagy at a late stage

**Fig. 1:**
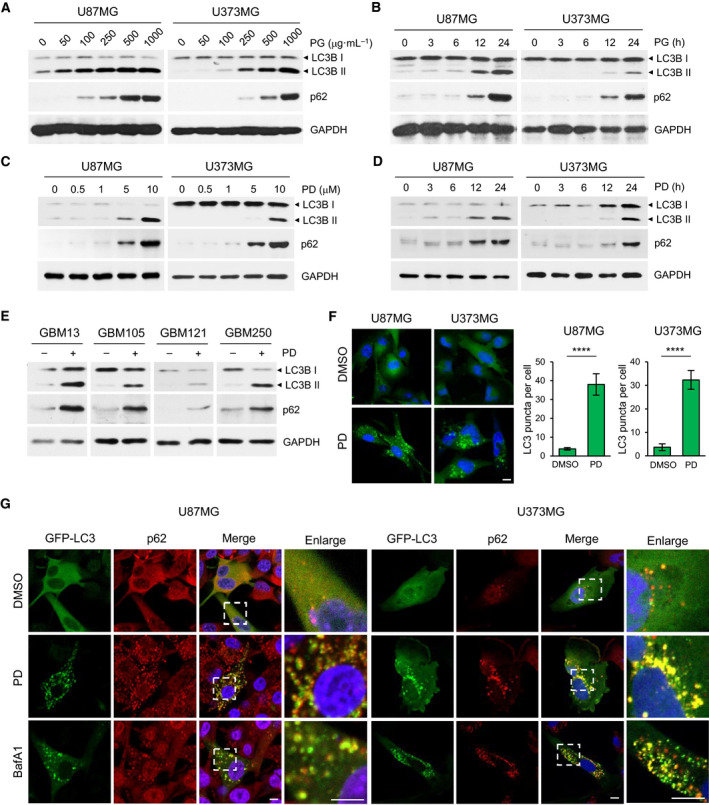
Platycodin D, a saponin present in *Platycodon grandiflorus*, increases levels of LC3B‐II and p62 in GBM cells. (A–E) U87MG and U373MG cells were incubated with indicated concentrations of PG extracts for 24 h (A) or incubated with 500 μg·mL^−1^ PG over a certain time period (3, 6, 12, and 24 h) (B). U87MG and U373MG cells were incubated with PD whose concentrations ranged from 0 to 10 μm for 24 h (C) or subjected to treatment of 10 μm PD for 3–24 h (D). Four different primary GBM cells were treated with 10 μm PD for 24 h (E). The levels of LC3B and p62 were determined by western blot analysis. GAPDH was used as a loading control. (F) U87MG and U373MG cells transiently expressing GFP‐LC3 were treated with DMSO or 10 μm PD for 24 h and analyzed by confocal microscopy. Nuclei were stained with DAPI (blue). Quantification shown on the right graph represents mean GFP‐LC3B puncta per cell from three independent experiments. Error bars indicate SD. Statistical differences were determined by unpaired, two‐tailed Student's *t*‐test. ****P < 0.0001. (G) U87MG and U373MG cells expressing GFP‐LC3B are treated with DMSO, 10 μm PD or 100 nm bafilomycin A1 (BafA1) for 24 h, followed by staining with anti‐p62 antibody and DAPI (blue). The merged images (yellow) indicate colocalization between LC3 and p62. Panels on the right are higher‐magnification images of the boxed regions. Scale bars: 10 μm in F and G.

To confirm the effect of PG extract on autophagy inhibition, U87MG and U373MG cells were treated with serial concentrations of PG from 50 to 1000 μg·mL^−1^ for 24 h and examined the levels of LC3B‐II and p62 by western blot. The results showed that both LC3B‐II and p62 were increased in a dose‐dependent manner (Fig. 1A) and reached a maximum at 24 h after treatment of 500 μg·mL^−1^ of PG in both GBM cells (Fig. 1B). Because PD, a triterpenoid saponin, is known as a major constituent of PG, we decided to test whether PD can exert inhibitory effects on autophagy. In GBM cells treated with varying concentrations of PD for 24 h, the level of both LC3B‐II and p62 was increased in dose‐dependent manner, showing the maximum activity at 10 μm PD (Fig. 1C). Time‐dependent increases in LC3B‐II and p62 were also observed, when the GBM cells were treated with 10 μm PD (Fig. 1D), indicating that PD is a phytochemical constituent of PG with autophagy‐inhibiting activity. Moreover, we found that PD increased the levels of both LC3B‐II and p62 in four different primary GBM cells, eliminating possibilities of cell line artifacts (Fig. 1E). Numerous studies have suggested that PD functions as an autophagy inducer in various cancers, such as lung and hepatocellular carcinoma [15]. However, these studies observed only LC3B‐II levels, but not p62 levels. To clarify the general role of PD in autophagy, we examined that effects of PD on p62 levels in various types of cancer cells including A549, H358, MCF7, HT29, and HepG2. Remarkably, the levels of p62 together with LC3B‐II were increased upon treatment with PG and PD in all tested cancer cells in a dose‐dependent manner (Fig. S2), providing evidence that PD is a *bona fide* autophagy inhibitor.

Next, the effects of PD on autophagy were determined by analyzing the formation of GFP‐LC3 labeled autophagosomes. Confocal microscopy showed that while the fluorescent signal of GFP‐tagged LC3 was visualized to be diffuse in the DMSO‐treated control cells, PD treatment markedly increased the formation of GFP‐LC3 puncta in both GBM cells (Fig. 1F). Quantification of GFP‐LC3 puncta per cell showed that 10.1‐fold increase (3.76 ± 0.66 in DMSO vs 38.03 ± 5.75 in PD) in U87MG cells and 8.9‐fold increase (3.68 ± 1.45 in DMSO vs 32.84 ± 3.98 in PD) in U373MG cells. p62 serves as an adaptor protein for mediating binding and degradation of ubiquitinated proteins in autophagosomes. Therefore, autophagy inhibition leads to accumulation of p62 and ubiquitinated proteins [16]. Staining of GBM cells expressing GFP‐LC3 with anti‐p62 antibody revealed that PD induced accumulation of p62 and its colocalization with GFP‐LC3 puncta in both GBM cells (Fig. 1G). Similar results were obtained when the cells were treated with bafilomycin A1 (BafA1), a well‐known autophagy inhibitor (Fig. 1G). On the other hand, PD treatment did not alter *p62* mRNA level in both GBM cells. (Fig. S3). These data demonstrate that the increased p62 levels by PD are mediated by blockade of p62 degradation, supporting the notion that PD inhibits autophagy at a late stage.

### 3.3 PD‐mediated autophagy regulation is independent of mTOR and MAPK signaling pathway

Previous studies have shown that PD modulates autophagy via PI3K/AKT/mTOR or MAPK pathway in human lung and liver cancer [19, 20]. Thus, we explored whether these signaling pathways may mediate the PD action on autophagy in GBM cells. Our results indicated that there were no significant changes in phosphorylation of AKT and S6K, a downstream effector of mTOR in the PD‐treated GBM cells (Fig. S4A). In addition, PD had no effect on phosphorylation of ULK‐1 at Ser‐757 and expression levels of ULK‐1, Beclin‐1, and ATG7 (Fig. S4A). We further found that PD did not change JNK phosphorylation in both GBM cells but increased ERK phosphorylation at 10 μm in U373MG cells (Fig. S4B). To assess the role of the increased phosphorylation of ERK in autophagy regulation, PD98059, a potent inhibitor of MEK, which is the upstream activator of ERK, was added before PD treatment and examined its effect on changes in LC3B‐II and p62 levels. While PD98095 effectively abrogated phosphorylation of ERK, it did not alter levels of LC3B‐II and p62 in U373MG cells (Fig. S4C), indicating that ERK activation in the PD‐treated U373MG cells is not related to autophagy regulation. Collectively, these data suggest that PI3K/AKT/mTOR or MAPK pathway is not involved in the PD‐mediated autophagy regulation in GBM cells, which was inconsistent with the previous results observed in other types of cancer cells.

### 3.4 PD blocks the fusion of autophagosomes with lysosomes

**Fig. 2:**
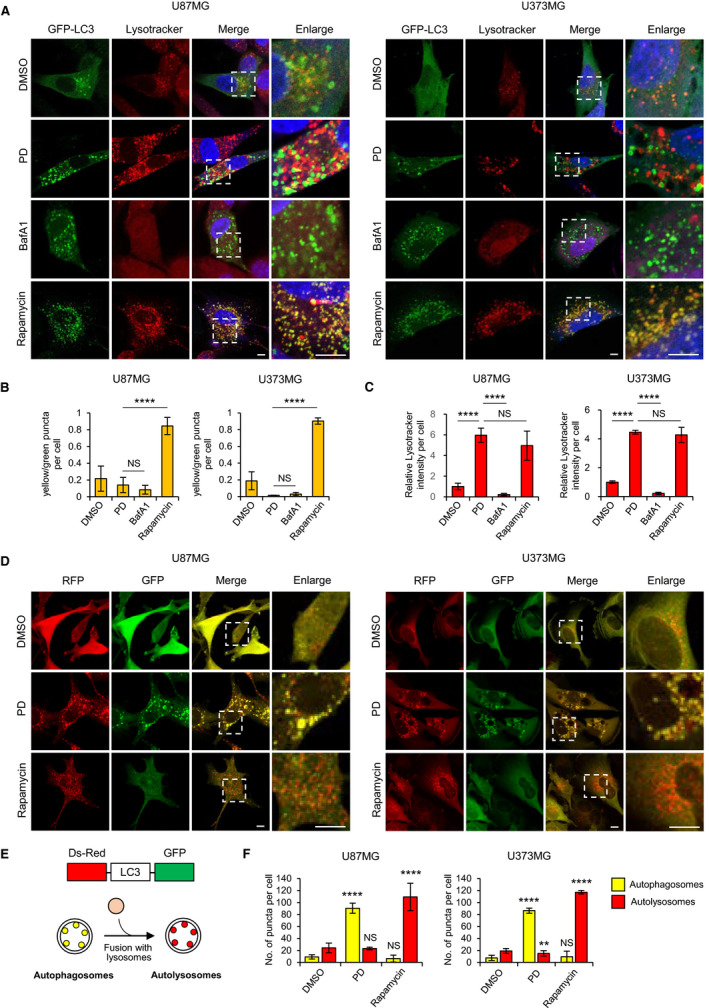
Platycodin D prevents autophagosome–lysosome fusion in GBM cells. (A) U87MG and U373MG cells transiently expressing GFP‐LC3 were exposed to 10 μm PD, 100 nm BafA1, or 1 μm rapamycin for 24 h and then stained with LysoTracker and DAPI (blue). The merged images show overlap of GFP‐LC3 and LysoTracker (yellow). Panels on the right are higher‐magnification images of the boxed regions. Scale bars: 10 μm. (B, C) Quantification of yellow and green puncta presented in individual cells was performed by manual counting. Autophagosome–lysosome fusion was estimated as the ratio between yellow and green puncta (B). The fluorescence intensity of LysoTracker was quantified with imagej software (C). 15–30 cells were counted in each condition. Error bars indicate SD. Statistical differences were determined by one‐way ANOVA using Tukey's *post hoc* test. ****P < 0.0001; NS, not significant. (B, C). (D) U87MG and U373MG cells stably expressing DsRed‐LC3‐GFP were incubated with 10 μm PD or 1 μm rapamycin for 24 h and then analyzed with confocal microscopy. The merged images (yellow) show overlap of GFP‐LC3 and DsRed‐LC3. Panels on the right are higher‐magnification images of the boxed regions. Scale bars: 10 μm (E) Diagram of Ds Red‐LC3‐GFP, an autophagic flux sensor. Autophagosomes appears yellow whereas autolysosomes appear red because GFP fluorescence is quenched under acidic environment after fusion with lysosomes. (F) Quantification of yellow and red puncta presented in individual cells was performed by manual counting. *N* > 20 cells. Error bars indicate SD. Statistical differences were determined by unpaired, two‐tailed Student's *t*‐test. **P < 0.01; ****P < 0.0001. NS, not significant.

To identify the mechanisms by which PD inhibits autophagy, we decided to explore the possibility that PD could impair autophagosome–lysosome fusion by staining the GBM cells expressing GFP‐LC3 with LysoTracker Red, a dye for labeling acidic compartments such as lysosomes. Intriguingly, confocal imaging showed that the GFP‐LC3 puncta generated by PD treatment were not colocalized with LysoTracker Red in both GBM cells, which were similar to those of cells treated with BafA1 that blocks lysosomal fusion with autophagosomes by inhibiting vacuolar H+ ATPase‐dependent lysosomal acidification [21] (Fig. 2A,B). In contrast, the treatment with rapamycin, an autophagy inducer, increased GFP‐LC3 puncta, which were largely colocalized with LysoTracker Red in both GBM cells (Fig. 2A,B). Notably, unlike BafA1, PD did not decrease but rather increased the LysoTracker Red fluorescence intensities, indicating that PD does not impair lysosomal acidification (Fig. 2A,C). We further examined the ability of PD to block the formation of the autolysosomes by inhibiting fusion of autophagosomes and lysosomes using a tandem‐tagged fluorescent reporter, DsRed‐LC3‐GFP. In the lysosomal acidic environment, the GFP fluorescence from the fusion protein is quenched but DsRed fluorescence in relatively stable [22]. In this respect, yellow puncta in the merged image are indicative of autophagosomes and solely red puncta correspond to autolysosomes (Fig. 2E). Confocal imaging showed that rapamycin treatment increased only DsRed puncta in GBM cells, indicating that GFP signal was attenuated in the acidic conditions of autolysosome. In contrast, PD treatment induced the formation of both GFP‐ and DsRed‐positive puncta and displayed yellow punctuate fluorescence, implying that PD compromised maturation of autophagosomes into autolysosomes (Fig. 2D,F). Taken together, these findings suggest that PD inhibits autophagy at a stage of autophagosome–lysosome fusion, which is not attributable to inhibition of lysosomal pH.

### 3.5 PD suppresses lysosomal proteolytic activity

**Fig. 3:**
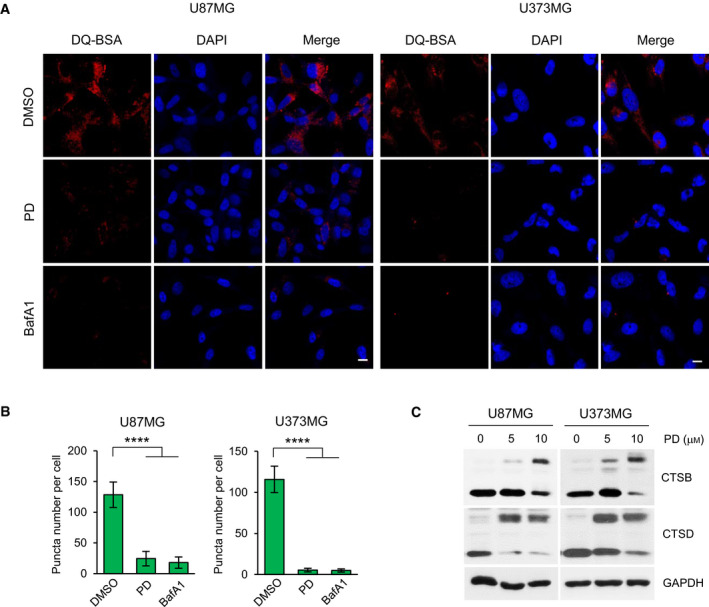
Platycodin D inhibits the lysosomal protease activity. (A) U87MG and U373MG cells were incubated with DMSO, 10 μm PD, or 100 nm BafA1 for 24 h before being treated with 10 μm DQ Red BSA for 30 min. Nuclei were stained with DAPI (blue). Data shown are representative images of each sample. Scale bars: 20 μm. (B) The fluorescence intensity of DQ‐BSA was quantified with imagej software. More than 100 cells were counted in each condition, and the number of puncta per cell is presented as means ± SD from three independent experiments. Statistical differences were determined by one‐way ANOVA using Tukey's *post hoc* test. ****P < 0.0001. (C) U87MG and U373MG cells were treated with 5 and 10 μm PD for 48 h. Western blot analysis for the processing of endogenous cathepsin B (CTSB) and cathepsin D (CTSD) is showed (upper, immature form/lower, mature form). GAPDH was used as a loading control.

Next, we investigate the effects of PD on lysosomal protease activity by DQ Red BSA assay. In DQ Red BSA, a red BODIPY dye conjugated to BSA, the BSA is so heavily conjugated that the red fluorescent is self‐quenched. Upon cleavage of the DQ Red BSA by proteolytic enzymes in lysosomes, this quenching is relived, generating red fluorescence. Thus, DQ Red BSA is a useful tool to visualize lysosomal proteolytic activity [23]. Confocal imaging revealed that PD caused a significant reduction in red fluorescence compared with the DMSO‐treated control in both GBM cells. Similar results were obtained in the BafA1‐treated GBM cells (Fig. 3A,B). Cathepsins are lysosomal cysteine proteases that are required for autophagy degradation of proteins engulfed into autophagosomes upon formation of the autolysosomes [24]. Procathepsins are cleaved to generate a mature 33 kD form that has been commonly used as a marker of measuring lysosomal activity. We evaluated the effects of PD on the processing of two lysosomal proteases including cathepsin B and cathepsin D. As shown in Fig. 3C, the active mature form of cathepsin B and cathepsin D was decreased and the inactive proform of those proteins was increased following PD treatment in both GBM cells. Collectively, these data demonstrate that PD disrupts lysosomal function via suppressing lysosomal proteolytic activity in GBM cells.

### 3.6 PD induces cholesterol accumulation in lysosomes, which leads to the impairment of autophagy in GBM cells

**Fig. 4:**
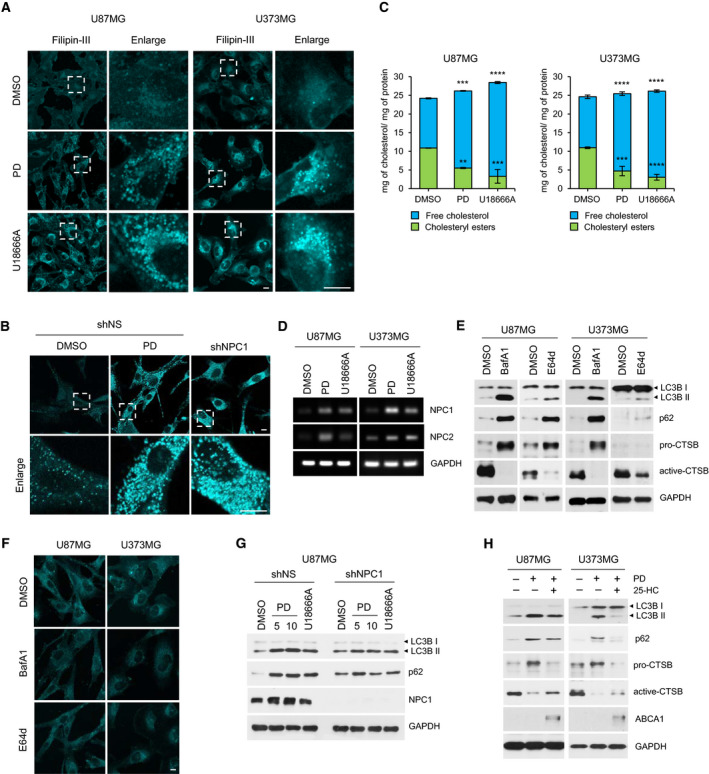
Platycodin D inhibits autophagy by promoting accumulation of cholesterol in lysosomes. (A, B) U87MG and U373MG cells were treated with DMSO, 10 μm PD, or 1 μm U18666A for 24 h (A). U87MG was infected with lentiviral particles containing nonspecific (NS) shRNA or NPC1 shRNA for 3 days, and then, the NS shRNA‐infected control cells were treated with DMSO or 10 μm PD for 24 h (B) Intracellular cholesterol distribution was determined by filipin‐III staining. Panels on the right are higher‐magnification images of the boxed regions. Scale bars: 10 mm. (C) The amount of free cholesterol and cholesteryl ester was measured by cholesterol assay kit. Total cholesterol levels were expressed in μg cholesterol per mg protein of cell lysate and shown as means ± SD from two independent experiments. Statistical differences were determined by one‐way ANOVA using Tukey's *post hoc* test. **P < 0.01; ***P < 0.001; ****P < 0.0001. (D) U87MG and U373MG cells were treated with DMSO, 10 μm PD, or 1 μm U18666A for 24 h, and *NPC1* and *NPC2* mRNA levels were analyzed by RT‐PCR. GAPDH was used as a loading control. (E, F) U87MG and U373MG cells were treated with 100 nm BafA1 or 10 μm E64d for 24 h. The expression of LC3B, p62, and cathepsin B was detected by western blot analysis. GAPDH was used as a loading control (E), and intracellular cholesterol distribution was determined by filipin‐III staining. Scale bars: 10 μm (F). (G) U87MG and U373MG cells were infected with lentiviral particles containing NS or NPC1 shRNA for 3 days and then treated with DMSO, 10 μm PD, or 1 μm U18666A for 24 h. The expression of LC3B, p62, and NPC1 was detected by western blot analysis. GAPDH was used as a loading control. (H) U87MG and U373MG cells were treated with 10 μm PD in the presence or absence of 10 μm 25‐hydroxycholesterol (25‐HC) for 24 h. The expression of LC3B, p62, cathepsin B, and ABCA1 was detected by western blot analysis. GAPDH was used as a loading control.

Platycodin D was reported to deplete intracellular cholesterol through activation of the liver X receptor (LXR)‐ATP‐binding cassette transporter A1 (ABCA1)‐mediated cholesterol efflux pathway in primary rat microglia cells [25]. Thus, we investigated whether the defects in autophagosome–lysosome fusion could be due to altered intracellular cholesterol levels. Intriguingly, staining the PD‐treated GBM cells with filipin‐III, a fluorescent dye that binds to free cholesterol, showed that PD caused a cholesterol accumulation within intracellular vesicles (Fig. 4A). Niemann‐Pick Type C (NPC) disease is characterized by massive accumulation of free cholesterol in the lysosomal compartment [26]. It is caused by loss‐of‐function mutations in either *NPC1* or *NPC2* gene, whose products mediate lysosomal egress of LDL‐derived cholesterol [27, 28]. U18666A is a cationic amphiphile that inhibit NPC1 function by directly binding to sterol‐sensing domain of NPC1 protein, phenocopying the effects of NPC1 mutation on intracellular cholesterol trafficking [29, 30]. In agreement with these studies, U18666A treatment of U87MG and U373MG cells or gene silencing of *NPC1* by shRNA in U87MG induced intracellular cholesterol deposits (Fig. 4A,B). Importantly, this punctate filipin‐III staining pattern was very similar to that of PD‐treated cells. Moreover, quantitative analysis of intracellular cholesterol revealed that free cholesterol levels were increased whereas cholesteryl ester, an intracellular storage form of cholesterol, was decreased almost identically in both PD‐ and U18666A‐treated GBM cells (Fig. 4C). We further found that *NPC1* and *NPC2* mRNA expression was upregulated after treatment of GBM cells with PD or U18886A, which could be as a result of compensatory response to the inhibition of NPCs (Fig. 4D). Inhibition of cathepsin B or L has been reported to cause NPC‐like cholesterol sequestration in human neuroblastoma [31]. To analyze whether the lysosomal cholesterol accumulation is due to the PD‐mediated inhibition of cathepsin B activity in GBM cells we observed in Fig. 3C, we monitored intracellular cholesterol distribution after treatment with BafA1 or E64d, cell‐permeable inhibitor of cysteine proteases. The results showed that the inactivation of cathepsin B by treatment with BafA1 or E64d blocked autophagy as assessed by the increased levels of LC3B‐II and p62 in western blot analysis (Fig. 4E) but did not lead to any changes in intracellular cholesterol distribution (Fig. 4F).

Next, we sought to investigate whether the lysosomal cholesterol deposits resulted in the inhibition of autophagy flux in GBM cells. Knockdown of NPC1 or U18666A treatment in U87MG cells increased the level of both LC3B‐II and p62, and no further augmentation of LC3B‐II and p62 was observed upon PD treatment in the NPC1 silenced cells (Fig. 4G). 25‐hydroxycholesterol (25‐HC), an endogenous oxysterol that acts as an agonist for LXR, blocks *de novo* cholesterol synthesis and promotes cholesterol efflux by induction of ABCA1 expression [32, 33]. Moreover, previous observations indicated that 25‐HC preferentially reduced the lysosomal cholesterol in NPC cells [34, 35]. Consistently, ABCA1 expression was induced by adding 25‐HC in GBM cells (Fig. 4H). In addition, the increase in LC3B‐II and p62 by PD was reversed by cotreatment of 25‐HC, which were more pronounced in U373MG cells (Fig. 4H). Moreover, in this condition, cathepsin B activity was returned to near pretreatment levels in both GBM cells (Fig. 4H). Overall, these results suggest that PD induces the accumulation of cholesterol in lysosomes, which contributes to the inhibition of lysosomal protease activity and autophagy flux.

### 3.7 PD increases LDL‐C uptake via upregulation of cell surface LDLR in GBM cells

**Fig. 5:**
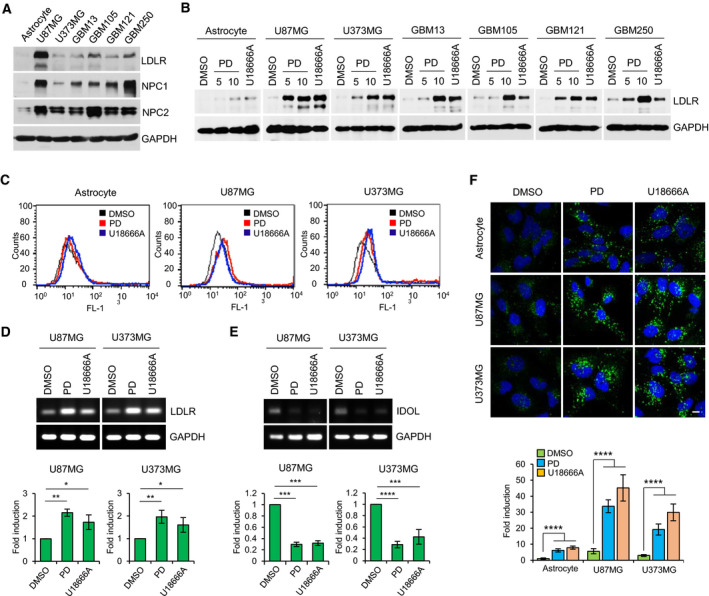
Platycodin D increases LDLR expression on cell surfaces in GBM cells. (A) Relative expression of LDLR, NPC1, and NPC2 in normal human astrocyte and GBM cells was detected by western blot analysis. GAPDH was used as a loading control. (B) Two GBM cell lines and four primary GBM cells were treated with DMSO, 5, 10 μm PD, or 1 μm U18666A for 24 h, and then, the protein levels of LDLR were assessed by western blot analysis. GAPDH was used as a loading control. (C) Normal human astrocyte and U87MG and U373MG cells were treated with DMSO, 10 μm PD, or 1 μm U18666A for 24 h, and the levels of cell surface LDLR were assessed by flow cytometry analysis. Representative histogram showed intensity of LDLR staining. (D, E) Relative mRNA levels of *LDLR* (D) and *IDOL* (E) were analyzed by RT‐PCR in U87MG and U373MG cells treated 10 μm PD or 1 μm U18666A for 24 h. GAPDH was used as a loading control. The figures show a representative gel of the RT‐PCR results. The intensity of *LDLR* (D) and *IDOL* (E) mRNA was determined using imagej software and normalized to that of GAPDH. Bar graph represents means ± SD from three independent experiments. Statistical differences were determined by one‐way ANOVA using Tukey's *post hoc* test. *P < 0.05; **P < 0.01; ***P < 0.001; ****P < 0.0001. (F) Normal human astrocyte and GBM cells were treated with DMSO, 10 μm PD, or 1 μm U18666A for 24 h and subsequently loaded with BODIPY™ FL LDL. BODIPY‐LDL uptake was visualized by confocal microscopy. Nuclei were stained with DAPI (blue). Scale bars: 10 μm. The fluorescence intensity was quantified with imagej software and presented as a relative fold change from three independent experiments and presented as means ± SD. Statistical differences were determined by one‐way ANOVA using Tukey's *post hoc* test. ****P < 0.0001.

**Fig. 6:**
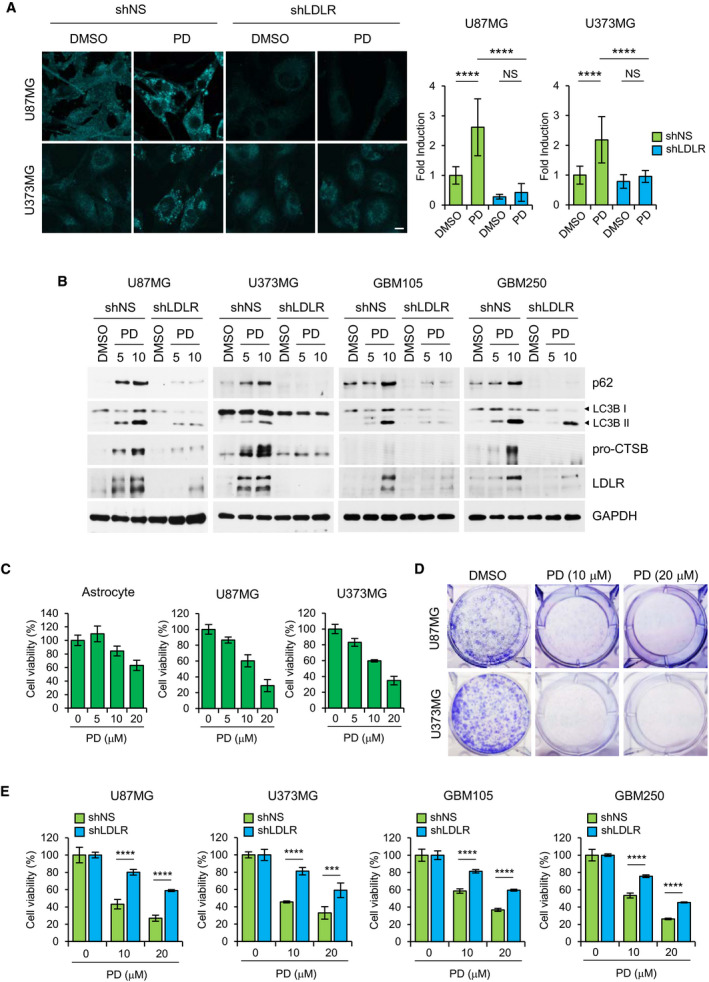
Low‐density lipoprotein receptor knockdown abolishes the effects of PD on autophagy inhibition and reduction in cell viability in GBM cells. (A, B) Where indicated, GBM cells were infected with lentiviral particles containing nonspecific (NS) shRNA or LDLR shRNA for 3 days and then treated with the indicated concentration of PD for 24 h. The cells stained with filipin‐III, followed by confocal imaging. Scale bars: 10 μm. The fluorescence intensity of filipin‐III was quantified with imagej software and presented as a relative fold change from two independent experiments. Error bars indicate SD. Statistical differences were determined by one‐way ANOVA using Tukey's *post hoc* test. ****P < 0.0001; NS, not significant (A). The expression of LC3B, p62, cathepsin B, and LDLR was detected by western blot analysis (B). (C) Normal human astrocyte and GBM cells were treated with the indicated concentration of PD for 48 h, and the relative cell viability was determined by WST1 assay. Results are presented as percentage relative to DMSO‐treated control. (D) U87MG and U373MG cells were treated with the indicated concentration of PD for 7 days and processed for clonogenic assay to measure proliferation ability of GBM cells. (E) GBM cells (shNS and shLDLR) were treated with the indicated concentration of PD for 48 h, and the relative cell viability was determined by WST1 assay. In the bar graphs, results are presented as percentage relative to DMSO‐treated control and represent means ± SD from two independent experiments. Statistical differences were determined by unpaired, two‐tailed Student's *t*‐test. ***P < 0.001, ****P < 0.0001.

Brain is the most cholesterol‐rich organ of the body, containing about 20% of total body cholesterol [36]. Previous studies demonstrated that GBM cells, in contrast to normal astrocytes which rely primary on the *de novo* synthesis of cholesterol, depend on the uptake of exogenous cholesterol via LDLR for their survival, thereby LDLR expression is upregulated in GBM cells [8, 9]. In consistent with these reports, we detected that LDLR expression was markedly elevated in two GBM cell lines and a panel of four primary GBM cells relative to normal astrocytes (Fig. 5A). We further observed that the protein levels of both NPC1 and NPC2 were significantly higher in GBM cells than normal astrocyte, implicating higher burden of processing LDL‐C in lysosomes in GBM cells (Fig. 5A). It was reported that sequestration of free cholesterol within lysosomes by U18866A leads to a decrease in endoplasmic reticulum (ER) cholesterol levels, resulting in induction of LDLR expression [37]. Therefore, we reasoned that PD might also enhance LDLR expression in GBM cells. Supporting this hypothesis, we found that both PD and U18666A significantly increased LDLR expression in all treated cells including two GBM cell lines and four primary GBM cells and normal astrocytes, but the increased levels of LDLR were more prominent in GBM cells than normal astrocytes (Fig. 5B). We next determined cell surface expression of LDLR by flow cytometry. U87MG and U373MG cells treated with PD or U18666A showed a shift of the FL‐1 histogram peak toward right with a similar extent, indicating that PD and U18666A increased LDLR expression on cell surface (Fig. 6C). However, the peak shift was very minor in normal astrocyte, which was equivalent to the results obtained by western blot analysis (Fig. 5B,C).

Next, we attempted to unveil the molecular mechanism of LDLR upregulation in the PD‐treated GBM cells and found that PD increased mRNA expression of *LDLR* (Fig. 5D) and decreased mRNA expression of inducible degrader of LDLR (*IDOL*), encoding an E3 ubiquitin ligase that targets LDLR for its lysosomal degradation (Fig. 5E). The reduction in *IDOL* expression by PD in GBM cells is consistent with what we recently discovered in hepatic cells [38]. Moreover, similar results were obtained in the U18666A‐treated GBM cells (Fig. 5D,E). Given that PD increases cell surface LDLR expression, we investigated the effects of PD on uptake of exogenous LDL‐C. Cells pretreated with PD or U18666A were incubated with BODIPY FL‐labeled LDL particles for 1 h and observed their uptake by confocal microscopy. Upon analysis of these cells, we detected that PD caused a marked increase in the uptake of LDL particles and it was a similar extent as those detected in the U18666A‐treated cells in both GBM cells (Fig. 5F). Of note, despite the increased rate of LDL‐C uptake was almost equal between the PD‐ or U18666A‐treated GBM cells and normal astrocyte, the total amount of LDL particles that entered cells was significantly higher in the drug‐treated GBM cells than in normal astrocytes (Fig. 5F). Taken together, these results demonstrate that PD increases cell surface LDLR expression and accelerates exogenous LDL‐C uptake, which is more prominent in GBM cells compared with normal astrocytes.

### 3.8 LDLR promotes PD‐mediated inhibition of autophagy and cell viability in GBM cells

We hypothesized that the increased uptake of exogenous cholesterol through elevated LDLR expression by PD might accelerate accumulation of cholesterol in lysosomes, thereby leading to the inhibition of autophagy and cell viability in GBM cells. To this end, we first silenced LDLR expression in GBM cells using lentiviral shRNA transduction. Knockdown of LDLR expression markedly prevented the PD‐mediated cholesterol uptake (Fig. S5) and the formation of cholesterol deposits in lysosomes (Fig. 6A) in both GBM cells. Importantly, silencing of LDLR in GBM cells reversed the PD‐induced increases in LC3B‐II and p62 levels as well as the inactivation of cathepsin B (Fig. 6B). Similar effects were observed in two primary GBM cells such as GBM105 and GBM250 that showed higher levels of LDLR expression than other two primary GBM cells (Fig. 5A). Next, we evaluated the effects of PD on GBM cell viability. PD at 10 μm, a concentration that effectively inhibited autophagy, reduced cell viability by approximately 40% after 2 days of treatment (Fig. 6C) and abolished colony formation capacity after 7 days of treatment in both U87MG and U373MG cells (Fig. 6D). Of note, PD was less toxic to normal astrocytes (Fig. 6C). More importantly, this growth‐inhibitory effect of PD was considerably blocked by LDLR knockdown in all tested GBM cells (Fig. 6E). Additionally, similar to LDLR knockdown, depletion of cellular cholesterol by culturing GBM cells in lipoprotein‐depleted serum (LD‐FBS) abrogated the decrease in cell viability induced by PD in U87MG and U373MG cell (Fig. S6). Collectively, these results demonstrate that the increased cholesterol uptake via LDLR and subsequent lysosomal cholesterol accumulation by PD lead to autophagy inhibition and GBM cell death.

## 4 Discussion

Autophagy pathway enables cancer cells to survive under metabolic stress by degrading cellular components and reusing them to meet their energy requirements. Therefore, suppression of autophagy is considered to be a therapeutic strategy to promote cancer cell death [39, 40]. Recently, numerous studies have explored the effect of PD on autophagy in various types of cancer but only observed the change in LC3‐II levels [19, 41]. Since LC3‐II levels can be increased not only by autophagy induction but also by a block in the later stages of autophagy, p62 levels should be evaluated to establish whether a compound may either induce or inhibit autophagy [16]. In this study, we conducted a screen of 30 traditional medicinal herbs to identify novel autophagy regulators and found that PG and its bioactive compound, PD elevated both LC3B‐II and p62 levels in GBM cells, and these changes were also observed in various different types of cancers including lung, colon, liver, and breast cancer, suggesting that PD might act as an general inhibitor of autophagy. We further explored the possibility of PD to inhibit autophagy using a combination of biochemical and cellular approaches in GBM cells. First, we demonstrated that PD inhibited autophagosome–lysosome fusion as measured by co‐imaging GFP‐LC3 with LysoTracker Red and probing autophagy flux with DsRed‐LC3‐GFP construct. Moreover, we found that PD impaired lysosomal degradation activity through analyzing the effect of PD on cathepsin B/D proteolytic maturation by western blot analysis and DQ Red BSA assay. Overall, our results support the notion that PD is a *bona fide* autophagy inhibitor.

Cholesterol is critically important for brain function, especially required for forming myelin sheaths that surround the axons in neurons [42]. Most of the cholesterol in our body is produced from liver, but it cannot be transferred to brain since blood–brain barrier blocks its transport from blood [43]. Therefore, most of cholesterol needed by neurons is synthesized and delivered in the form of LDL particles from neighboring astrocytes [44]. Through LDLR‐mediated endocytosis, LDL‐derived cholesteryl esters are transferred to lysosomes where it is hydrolyzed to unesterified, free cholesterol by lysosomal acid lipase (LAL) and mobilized by two lysosomal cholesterol transporters such as NPC1 and NPC2 [45, 46] to other cellular organelles such as plasma membrane, ER, and mitochondria [47]. Therefore, loss of function of these proteins leads to the massive accumulation of free cholesterol in lysosomes. An important observation made in this study is that PD promotes an accumulation of cholesterol within intracellular vesicles and increases the portions of free cholesterol, which were almost identical to those in the cells treated with an NPC1 inhibitor, U18666A. These results suggest that PD inhibits intracellular cholesterol trafficking, which might be associated with defects in cholesterol transport processes from lysosomes to other organelles. A key question that arises from these results is whether the defective autophagy caused by PD is attributed to cholesterol accumulation in lysosomes. Fraldi *et al*. [48] demonstrated that excessive cholesterol in lysosomal membrane interfered with proper assembly and recycling of soluble *N*‐ethylmaleimide‐sensitive factor attachment protein receptors (SNAREs), key components of the intracellular membrane fusion machinery. These authors have also shown that reduction in cholesterol levels in the lysosomal membranes restored normal SNARE function and lysosomal fusion with autophagosomes. In line with these observations, prevention of LDL‐C accumulation in lysosomes by LDLR knockdown abolished the PD‐mediated inhibition of lysosomal protease activity and autophagy flux, indicating that the lysosomal accumulation of LDL‐C is a main cause of the PD‐mediated blockade of autophagy progression. A recent study reported that transport of autophagosomes toward perinuclear region and their fusion with lysosomes were prevented under low intracellular cholesterol levels [49]. The PD‐mediated sequestration of cholesterol in lysosomes may lead to reduction in cholesterol content in cytoplasm, which is supported by our results showing that PD elevates LDLR levels, which is upregulated in response to low intracellular cholesterol availability [12]. Therefore, the possibility cannot be ruled out that PD negatively impacts on autophagosome–lysosome fusion via induction of low levels of intracellular cholesterol.

**Fig. 7:**
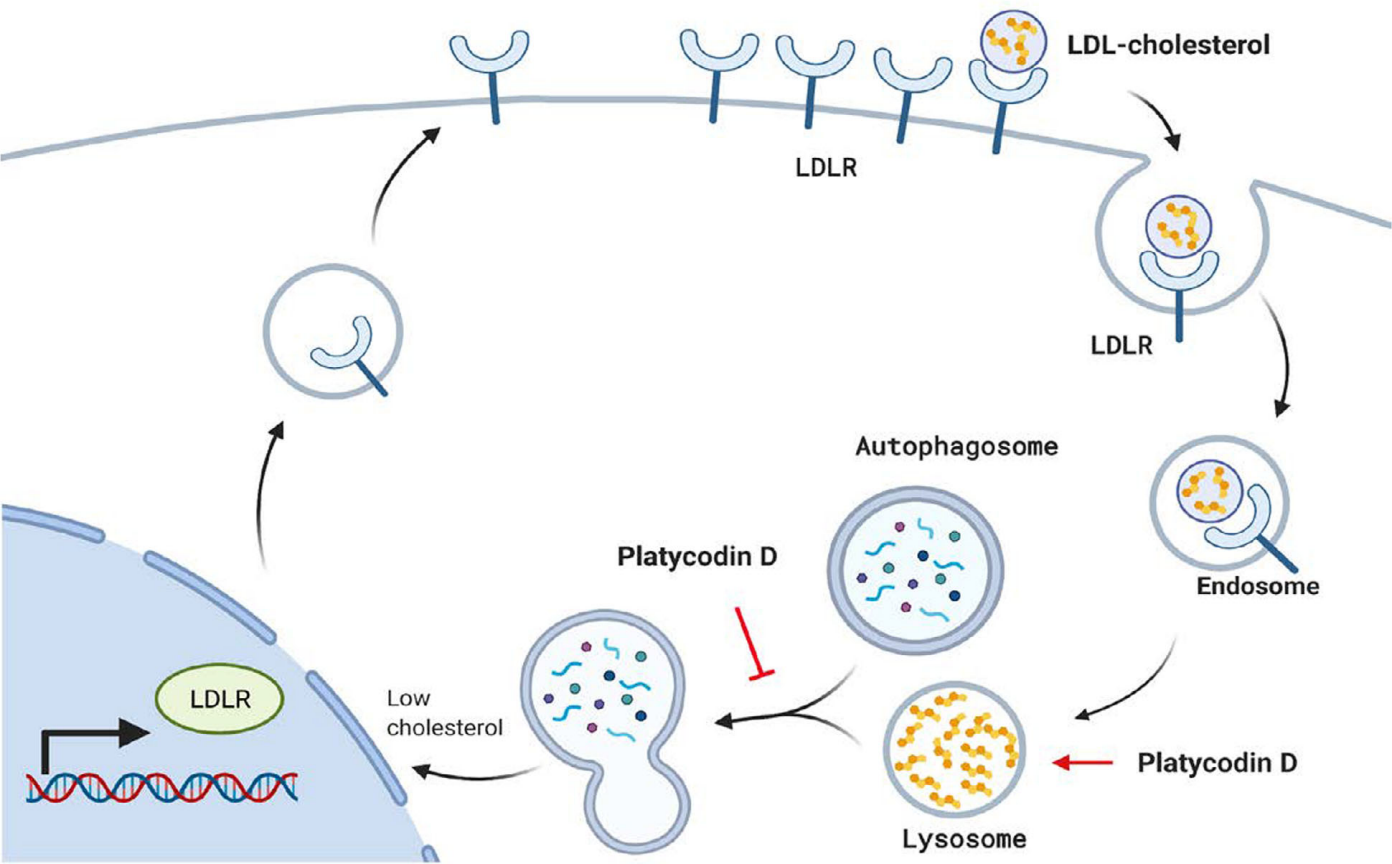
Proposed model of platycodin D‐mediated autophagy inhibition and cell death in LDLR‐overexpressing glioblastoma. Platycodin D increases cholesterol content in lysosomes, possibly by disrupting lysosomal cholesterol trafficking. The accumulation of cholesterol in lysosomes prevents lysosomal fusion with autophagosomes and decreases usable cholesterol levels. The low cholesterol availability makes cells increase LDLR expression and further uptake LDL cholesterol, but which causes a vicious cycle of continuous accumulation of cholesterol in lysosomes and leads to GBM cell death. This phenomenon is more evident in LDLR‐overexpressing GBM cells than normal astrocytes expressing low levels of LDLR.

Cholesterol metabolism has emerged as a novel therapeutic target for the treatment of GBMs [50]. Unlike normal astrocytes, GBM cells cannot produce cholesterol via its biosynthesis pathway, thus should uptake exogenous cholesterol through LDLR to meet cholesterol requirements for their growth [9]. The LDLR upregulation can be achieved by epidermal growth factor receptor (EGFR) signaling [8], which is mutated or amplified in around 50% of GBMs [51]. In agreement with these findings, we found that GBM cell lines and primary GBM cells exhibited elevated levels of LDLR compared with normal astrocytes. These unique features render GBM cells addicted to exogenous cholesterol supply for their survival and more susceptible to cell death upon cholesterol depletion by treatment with LXR agonists that stimulate ABCA1‐dependent cholesterol efflux and inhibit cholesterol uptake by promoting the IDOL‐mediated LDLR degradation [8, 9, 52]. Contrary to these finding, PD elevated intracellular cholesterol levels by increasing uptake of LDL particles via LDLR upregulation. However, PD may not able to increase the levels of usable cholesterol in cells because PD sequestered them in lysosomes. This isolation of cholesterol in lysosomes drives further uptake of LDL‐C, which may lead to a vicious cycle of continuous accumulation of cholesterol in lysosomes (Fig. 7). Importantly, PD displayed less cytotoxic effect on normal astrocyte compared with GBM cells. We reasoned that this might be associated with lower levels of LDLR expression in normal astrocytes relative to those in GBM cells. This hypothesis was also supported by our results showing that knockdown of LDLR in GBM cells reduced the impact of PD on GMB cell death, notably which was similar to the cell viability that was seen in the PD‐treated normal astrocytes (Fig. 6C,E).

What is the primary molecular target of PD activity? PD exhibits a very similar phenotypic effects with U18666A, raising possibility that PD and U18666A share common molecular target. U18666A is structurally similar to cholesterol and known to directly bind to sterol‐sensing domain of NPC1 [29]. At the level of chemical structure, PD consists of a pentacyclic triterpene with a sugar and oligosaccharide moiety on each sides. Since the triterpene aglycone of PD resembles U18666A and cholesterol, PD is likely to interact with the same site of NPC1 where U18666A binds. Further studies are needed to demonstrate the direct interaction between PD and purified NPC1 using modern technologies such as surface plasmon resonance or isothermal titration calorimetry.

## 5 Conclusion

In summary, our findings suggest that PD induces GBM cell death by inhibiting autophagy flux, which is attributed to the accumulation of LDL cholesterol in lysosomes. We further demonstrate that the anticancer effect of PD correlates with a high abundance of LDLR in GBM cells. Therefore, we propose that PD can be a potential anti‐GBM drug that targets autophagy and cholesterol metabolism on which GBM cells depend for survival.

## Conflict of interest

The authors declare no conflict of interest.

## Author contributions

TYK and S‐GK conceived and designed the experiments. SJL, TYK, Y‐JC, and HIK conducted experiments. HEM and SHP produced primary GBM cells. SJL and TYK analyzed the data and wrote, reviewed, and edited the manuscript. S‐GK supervised the research.

### Peer Review

The peer review history for this article is available at https://publons.com/publon/10.1002/1878‐0261.12966.

## Supporting information


**Fig. S1.** Screening of various traditional herbal medicines for identifying novel autophagy regulators.
**Fig. S2.** PG and PD increases levels of LC3B‐II and p62 in various types of cancer.
**Fig. S3.** PD does not increase p62 mRNA levels.
**Fig. S4.** PD‐mediated autophagy regulation is independent of mTOR and MAPK signaling pathway.
**Fig. S5.** LDLR knockdown abolishes the effects of PD on LDL uptake in GBM cells.
**Fig. S6.** Cholesterol depletion abrogates PD's inhibitory effect on cell viability in GBM cells.

## Data Availability

The data that support the findings of this study are available from the corresponding author upon reasonable request.
