# Supplementary material for: Platycodin D inhibits autophagy and increases glioblastoma cell death via LDLR upregulation
Source: Mol Oncol. 2021 May 2;16(1):250–68. doi: 10.1002/1878-0261.12966 (PMC8732342; doi:10.1002/1878-0261.12966)
Supplement: Supplementary file 1 — Fig. S1. Screening of various traditional herbal medicines for identifying novel autophagy regulators. Fig. S2. PG and PD increases levels of LC3B‐II and p62 in various types of cancer. Fig. S3. PD does not increase p62 mRNA levels. Fig. S4. PD‐mediated autophagy regulation is independent of mTOR and MAPK signaling pathway. Fig. S5. LDLR knockdown abolishes the effects of PD on LDL uptake in GBM cells. Fig. S6. Cholesterol depletion abrogates PD's inhibitory effect on cell viability in GBM cells. [file MOL2-16-250-s001.pdf]

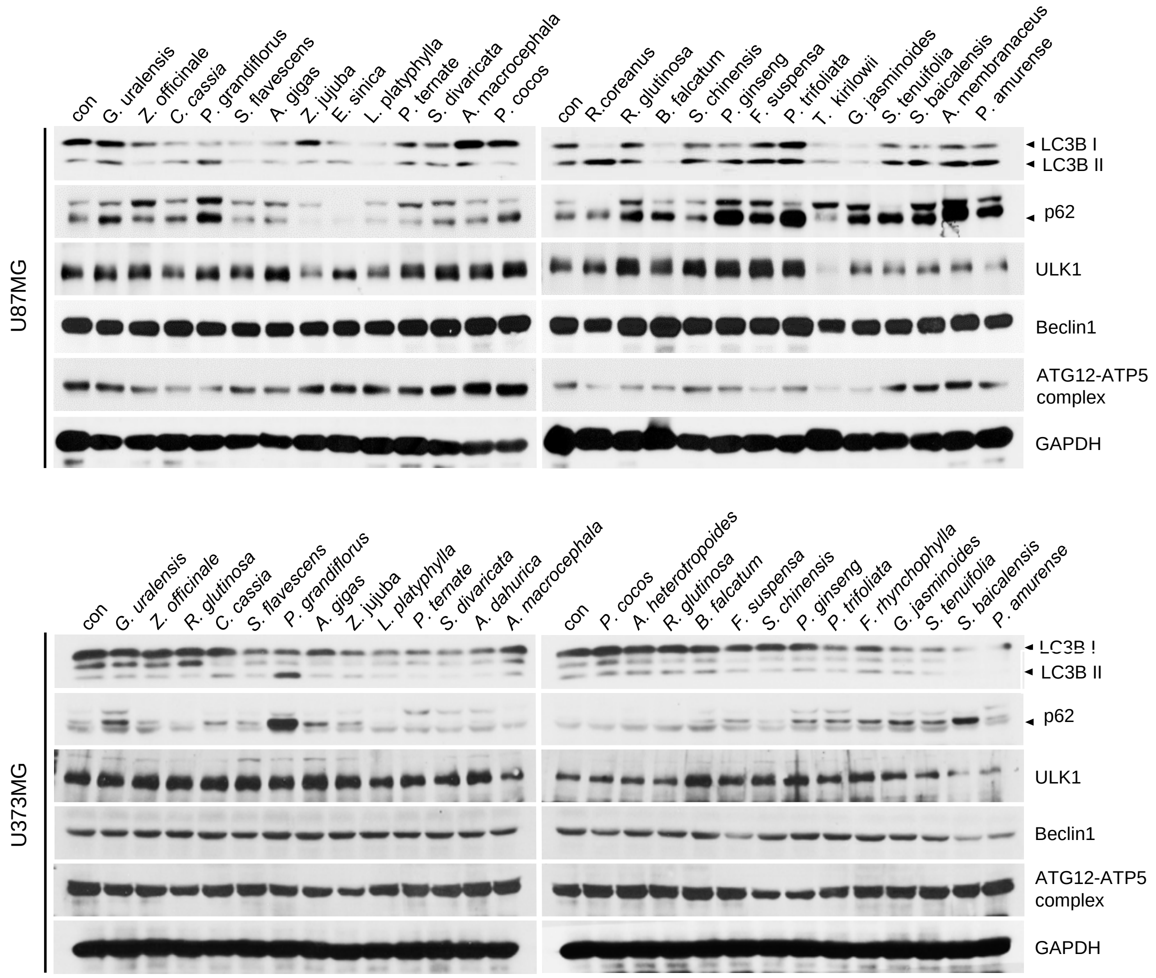

Fig. S1. Screening of various traditional herbal medicines for identifying novel autophagy regulators. Water extracts of 30 traditional medicinal herbs were treated to U87MG and U373MG cells at a concentration of 500 μg/ml for 48 h. The levels of LC3B, p62, ULK1, Beclin1, and ATG12-ATG5 complex in the whole cell lysates from the treated cells were evaluated by western blot analysis. GAPDH was used as a loading control.

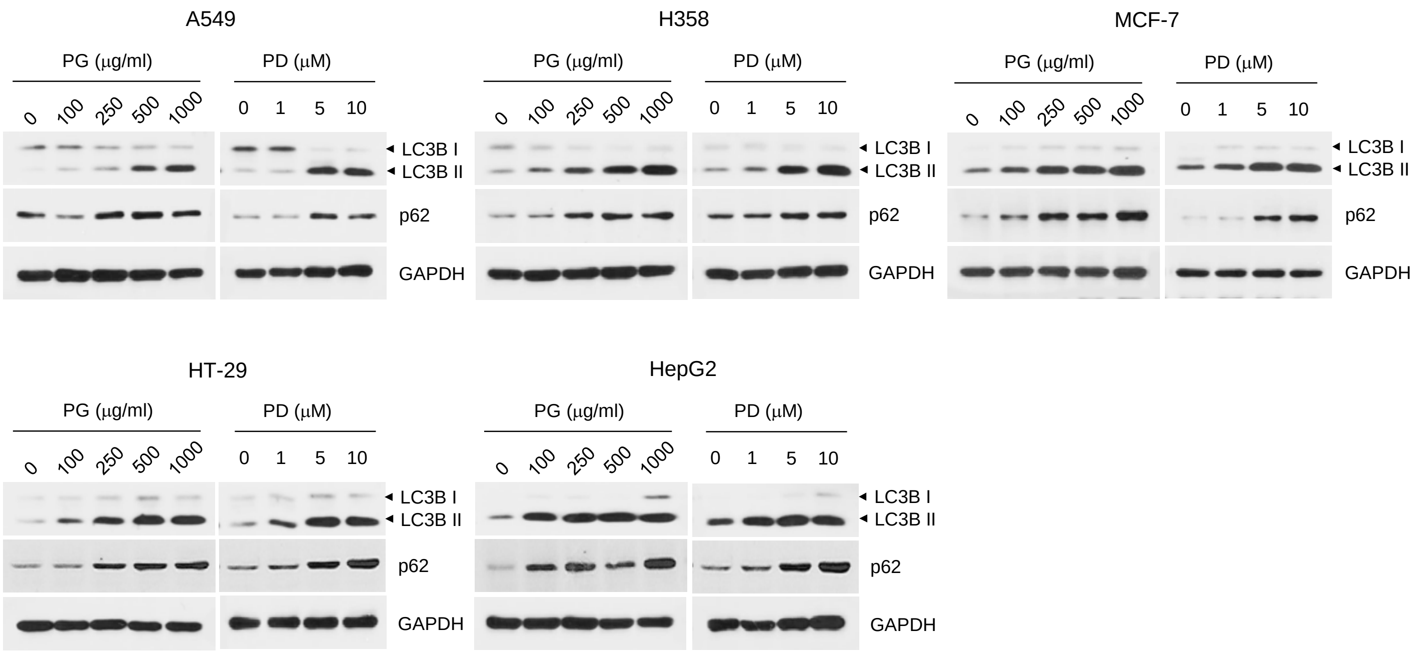

Fig. S2. PG and PD increases levels of LC3B-II and p62 in various types of cancer. After treatment with the indicated concentrations of water extract of PG or PD for 24 h, the levels of LC3B-II and p62 were detected by western blot analysis in several cancer cell lines including A549, H358 (lung cancer), MCF7 (breast cancer), HT29 (colon cancer) and HepG2 (liver cancer). GAPDH was used as a loading control.

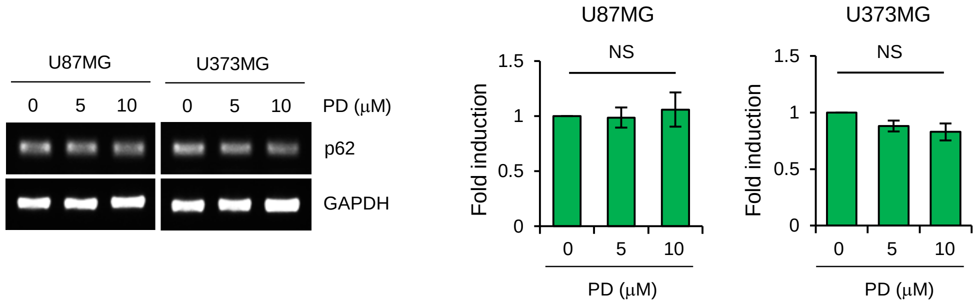

Fig. S3. PD does not increase *p62* mRNA levels. *p62* mRNA levels were analyzed by RT-PCR in U87MG and U373MG cells treated with DMSO or PD at a concentration of 5 and 10 μM for 24 h. GAPDH was used as a loading control. The figures show a representative gel of the RT-PCR results. The intensity of *p62* mRNA were determined using Image J software and normalized to that of GAPDH. Bar graph represents means ± SD from three independent experiments. Statistical differences were determined by one-way ANOVA using Tukey's post hoc test. NS, not significant.

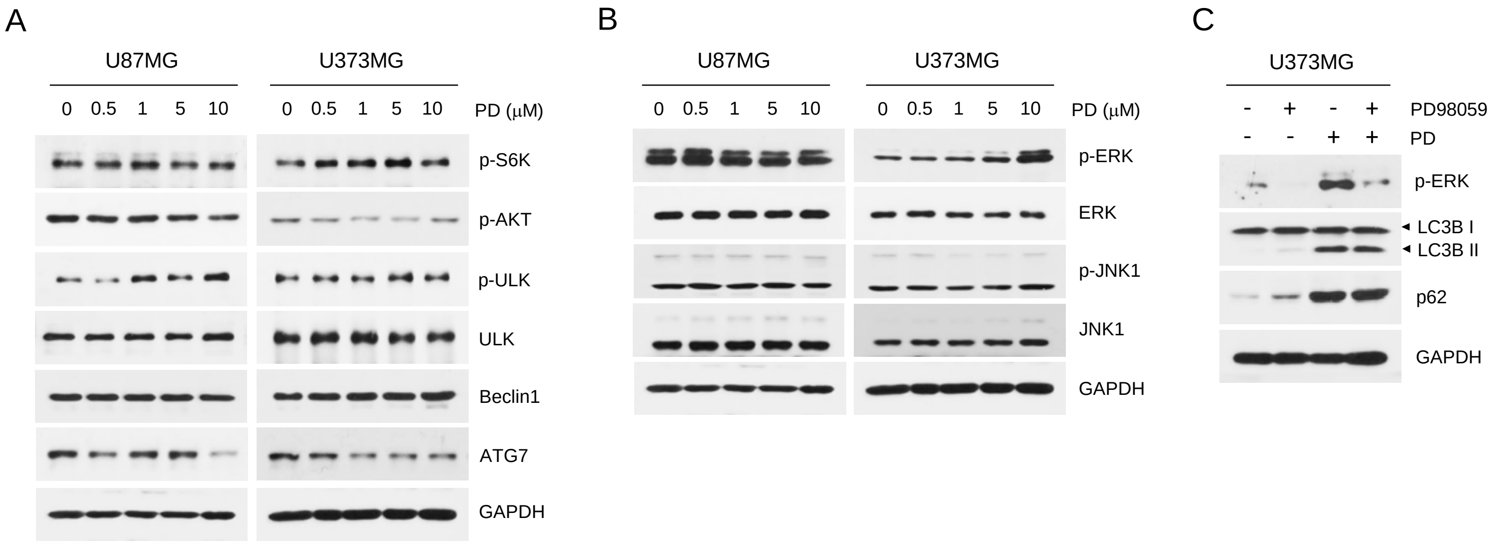

Fig. S4. PD-mediated autophagy regulation is independent of mTOR and MAPK signaling pathway. (A, B) U87MG and U373MG cells were treated with the indicated concentration of PD for 24r. Using lysates from the PD-treated GBMs, western blot analysis for p-ULK1 (Ser757), ULK1, Beclin1, ATG7, p-S6K (Thr389), p-AKT (Ser473), p-ERK, ERK, p-JNK1, and JNK1 was performed. GAPDH was used as a loading control. (C) U373MG cells were pre-incubated for 30 min with 50 µM PD98059, an inhibitor of the MEK/ERK pathway and then treated with 10 µM PD for 24 hr. Total protein extracts were analyzed by western blot analysis for p-ERK, LC3B, and p62. GAPDH was used as internal control.

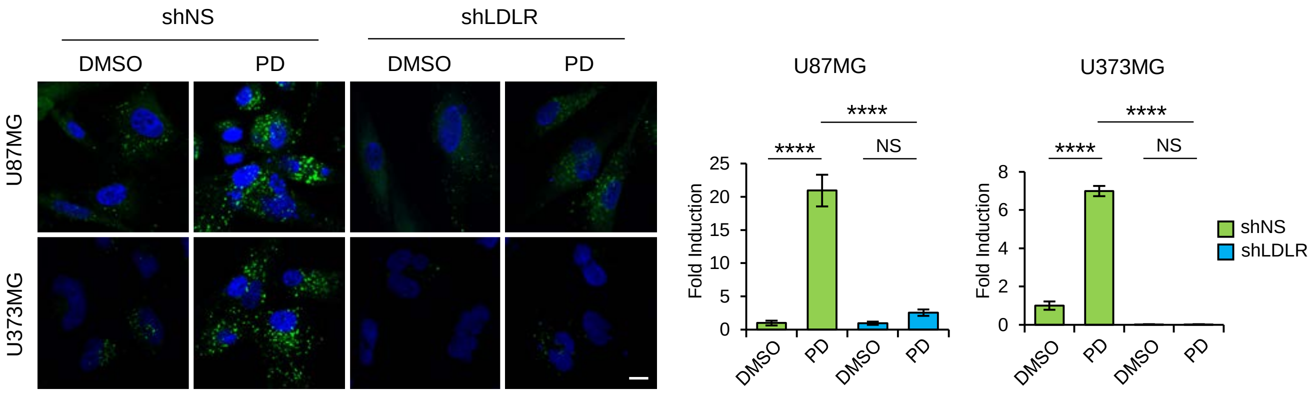

Fig. S5. LDLR knockdown abolishes the effects of PD on LDL uptake in GBM cells. U87MG and U373MG cells were infected with lentiviral particles containing NS shRNA or LDLR shRNA for 3 days and then treated with 10 μM PD for 24 h. The cells were loaded with BODIPY™ FL LDL, followed by confocal imaging. Scale bar: 10 μm. The fluorescence intensity was quantified with Image J software and presented as a relative fold change from three independent experiments. Error bars indicate SD. Statistical differences were determined by one-way ANOVA using Tukey's post hoc test. ****$P < 0.0001$; NS, not significant.

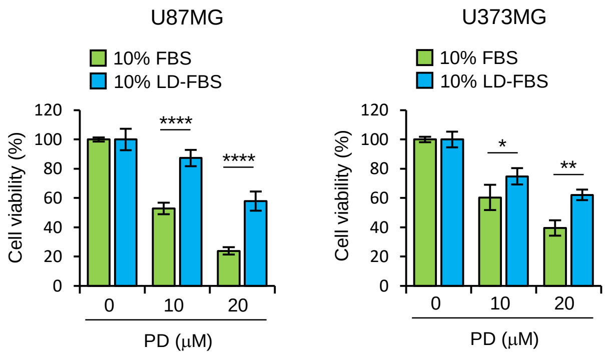

Fig. S6. Cholesterol depletion abrogates PD's inhibitory effect on cell viability in GBM cells. U87MG and U373MG cells were incubated in culture media containing 10% lipoprotein-depleted FBS (LD-FBS) for 2 days and then treated with the indicated concentration of PD for 2 days. Cell viability was assessed by WST1 assay. Results are presented as percentage relative to DMSO-treated control and represent means ± SD from two independent experiments. Statistical differences were determined by unpaired, two-tailed Student's t-test. *$P < 0.05$, **$P < 0.01$, ****$P < 0.0001$.
